# Ontologies for Bioinformatics

**DOI:** 10.4137/bbi.s451

**Published:** 2008-03-12

**Authors:** Nadine Schuurman, Agnieszka Leszczynski

**Affiliations:** Department of Geography, Simon Fraser University RCB 7123, 8888 University Drive, Burnaby, British Columbia, Canada, V5A 1S6

**Keywords:** ontologies, semantics, biological databases, bioinformatics, Gene Ontology

## Abstract

The past twenty years have witnessed an explosion of biological data in diverse database formats governed by heterogeneous infrastructures. Not only are semantics (attribute terms) different in meaning across databases, but their organization varies widely. Ontologies are a concept imported from computing science to describe different conceptual frameworks that guide the collection, organization and publication of biological data. An ontology is similar to a paradigm but has very strict implications for formatting and meaning in a computational context. The use of ontologies is a means of communicating and resolving semantic and organizational differences between biological databases in order to enhance their integration. The purpose of interoperability (or sharing between divergent storage and semantic protocols) is to allow scientists from around the world to share and communicate with each other. This paper describes the rapid accumulation of biological data, its various organizational structures, and the role that ontologies play in interoperability.

## Introduction

During the 1960s, there was a simultaneous evolution of digital protein and taxonomic inventories. By the 1980s, these had matured and were institutionalized with an attendant proliferation of biological data. These datasets were, however, maintained in closely-guarded proprietary repositories or ‘silos’ with little or no communication between them (Bisby, 2000; Boguski and McIntosh, 2003; Pennisi, 2000). The 1990s were marked by a shift in emphasis from accumulating vast volumes of data to reducing overlap between databases and making use of extant data across various repository locations (Boguski and McIntosh, 2003). This process of increasing communication between databases is known as interoperability—the focus of which is to enable data sharing and comparison.

As the cumulative body of biological knowledge increases, generating a comprehensive and consistent account of biology hinges upon the ability of scientists to draw upon and synthesize vast datasets across distributed digital resources. The ultimate objective of biodiversity informatics is to generate a “global inventory of [all] life on Earth” (Blackmore, 2002, p 365), and is premised on the seamless digital accumulation of distributed taxonomies. Because contemporary biological—particularly ‘omics’ and model organism—databases stress data at the molecular scale, they do not adequately represent the physiology they describe (Boguski and McIntosh, 2003). There is thus a need to compile the cellular features of those organisms into discernible representations of those organisms themselves.

The rise of ‘omics’ science—genomics, proteomics, and metabolomics for the identification and prediction of genetic product components, signatures, and processes (Sauer et al. 2007, p 550)—has contributed the molecular-level information upon which a systems view of biology is predicated. Certainly the complexity of biology resides at the level of gene products (Sauer et al. 2007). In this way biodiversity can be understood as the compendium of the biology of organisms (Chicurel, 2002b). In a computational environment, biodiversity is the hereditary information encapsulated within genetic products and identified via the collective of mappings of several model organism genomes. While the maturation of ‘omics’ has been facilitated in large part by the capacity to seamlessly make divergent data sources interoperable, it has presented a new set of engineering challenges. These include the need to integrate diverse and remote data sources as well as to extract knowledge from digital information post-integration (Thomas and Ganji, 2006).

The paradigm shift the ‘omics’ revolution has created within biology is best exemplified by gene prediction (also known as gene finding), and functional prediction tasks. New technologies such as micro arrays generate huge and ever-changing volumes of data (Buetow, 2005). The rapid growth of genome mapping necessitates the ability to automate gene-calling, or the identification of the individual genes of a genome. Gene finding involves algorithms for the identification of biologically functional regions—or exons—of sequences which explicitly code for proteins (Saeys et al. 2007). These are referred to as *coding regions*. The objective of automated gene prediction is thus to determine the “coding potential” of genetic sequences (Saeys et al. 2007, p 414). This process uses self-learning algorithms which predict unique signatures of the genetic spectrum that indicate distinct clusters of material (Boguski and McIntosh, 2003; Chicurel, 2002b). Where genes have been located, the biological functions of many protein sequences are as yet undetermined (Carroll and Pavlovic, 2006). Gene finding and functional prediction go hand in hand and are rarely treated separately as researchers often desire to discern the roles of newly identified gene products (Camon et al. 2004). The potential for predicting protein function similarly rests on its inference over incompletely annotated sequences on the basis of homologues in other species (Carroll and Pavlovic, 2006). However, neither is an easy feat as the coding regions of eukaryotic organisms are both sparse and small, making the identification of exon/intron boundaries—and thereby the identification of protein function—difficult, resulting in erroneous gene annotation (Saeys et al. 2007). In the present era of functional genomics, knowledge production is however dependent on the ability to recover genes and proteins on the basis of their (correctly) annotated functionality, pathways, and/or protein-protein interactions (Boguski and McIntosh, 2003). This is no trivial task; indeed it necessitates the resolution of *semantics*, or differences in meaning and naming conventions between distributed data resources (Bowker, 2000).

Unlike systems architectures, the integration of which constitutes an ‘IT problem’ (Searls, 2005), data are not semantically transparent. Although a structural linkage can now be easily defined between data sources such that a user can retrieve data on the basis of standardized queries across data sources with conflicting database schemas, this does not render the results of those queries meaningful. A prime example is the notion of ‘gene’—the primitive of modern biology. While the concept of ‘gene’ is still evolving, two dominant concepts exist: the Human Genome Database defines a gene as a DNA fragment that can be interpreted as (analogous to) a protein; whereas GenBank and the Genome Sequence Database (GSDB) consider a gene to be a “ ‘region of biological interest with a name and that carries a genetic trait’ ” (Schulze-Kremer, 2002, p 180). Two databases can be developed based on different understandings of ‘gene.’ As a result, retrieving data from semantically orthogonal databases on the basis of a ‘gene’ keyword search can initiate error propagation—in this case in the form of false analogues—in the analysis and subsequent results (Searls, 2005). The complexity of biological terms exacerbates this problem. Even where two variables in disparate databases are semantically equivalent, their relations to other knowledge objects in the data repository may not be. This is referred to as schematic incompatibility and refers to the relative position of the term in a taxonomic hierarchy.

In order to accommodate both semantic and schematic differences between biological databases, ‘omics’ research requires a method of expressing the *contexts* from which biological concepts emerge—at the database level. This is because functional prediction hinges upon the identification not just of sequence homologues but similar cellular components participating in a similar biological process. The component cellular, molecular, and biological details are often located in separate data sources, a function of the narrow scope of biological information produced by any given laboratory (Lewis, 2005). Exploiting the vast digital resources of biological data for prediction services requires that the cellular, molecular, and biological contexts of proteins be adequately encoded and furthermore machine-readable.

*Ontologies*—or the use of a singular taxonomic and knowledge representation schema—are a way of resolving these semantic issues between databases. The bioinformatics literature has been heavily promoting ontologies as an operational solution for biological interoperability since the turn of the millennium (Ashburner et al. 2000, p 25–9, Blake, 2004; Boguski and McIntosh, 2003; Buckingham, 2004a; Buckingham, 2004b; Buetow, 2007; Buetow, 2005; Camon et al. 2004; Carroll and Pavlovic, 2006; Castro et al. 2006; Chicurel, 2002a; Chicurel, 2002b; Galperin, 2006; Giles, 2007; Hill et al. 2002; Kohler et al. 2003; Kohler and Schulze-Kremer, 2002; Lewis, 2005; Peters and Sette, 2007; Schulze-Kremer, 2002; Searls, 2005; Wolstencroft et al. 2005). Much of this literature assumes that the reader has a prior understanding of computing and is delivered in impenetrable technical language or emphasizes a singular aspect of ontologies in biology.

The power of ontologies lies in their capacity to provide context for biological semantics. This paper presents the molecular biologist—rather than the computing scientist—with a detailed, comprehensive review of ontologies in biology. We begin with a definition of formal ontology in order to clarify the role that ontologies play with respect to interoperability (or the exchange of data). We describe ontological concepts—and their role bioinformatics—using the examples of two preeminent ontological efforts in biology: the Gene Ontology (GO), which is itself part of the umbrella Open Biomedical Ontologies (OBO) initiative. Subsequently we explain how ontologies can be exploited to facilitate information sharing and data integration efforts for bioinformatics with reference to real-world, large-scale biological information portals, namely the cancer Biomedical Informatics Grid (caBIG), and WikiProteins, a proprietary knowledge commons for proteins.

Furthermore, we describe a methodology for using ontologies as a basis for comparing semantics across health registries in order to illustrate how medical informaticians have imposed interoperability on disjunct datasets. Once semantic and schematic heterogeneity is resolved between data-sets, we explain how ontologies can be used to facilitate knowledge creation tasks in biology, such as automating gene/protein annotation and functional prediction.

To provide a global overview of ontologies for biology, we also draw upon a related community of research—health/medical informatics—which uses and shares with bioinformatics a series of knowledge representation constructs for the capture of biological information. Both use genetic information in the era of “ ‘post-genome’ science” (Boguski and McIntosh, 2003, p 233). For instance, knowledge sharing protocols developed in the field of health informatics are shared by bioinformatics researchers for resolving semantic heterogeneity in databases. Wang et al. (2005) for example use Protégé—an open-source ontology editing and knowledge acquisition software authored by Stanford Medical Informatics (2005)—as the knowledge representation platform for their mediation architecture. There are similarly links between bioinformatics and biodiversity communities. The tools of bioinformatics—many of which emerge from health informatics—are ideally suited to the objectives of biodiversity research, particularly conservation science (Sugden and Pennisi, 2000). This paper nevertheless emphasizes bioinformatics.

## Ontologies

In philosophy, ontology has traditionally been understood to be the essence of being—or what something really is (Schuurman, 2006). In the information sciences, an ontology is a fixed universe of discourse in which each element (e.g. field name or column in a database) is precisely defined (Gruber, 1993). In addition, each possible relationship between data elements is parametized or constrained. For example, DNA may comprise chromosomes but not the reverse. In an ontology, these relationships are made explicit formally.

The prefix ‘formal’ refers to the property of machine-readability (Agarwal, 2005). In other words, a *formal ontology* is a machine-readable model of the objects allowed into a formal universe and their associations or relationships between them upon which some automated reasoning tasks can be performed. In a formal environment, an ontology constitutes a surrogate of knowledge abstracted from the real world—in this case, the cumulative body of biological science—in a coded form that can be translated into a programming language (Smith et al. 2003; Sowa, 2000).

Scientific or systems ontologies contain three levels of formalization. The first is the conceptual, which is then translated into a formal model of the data elements in the ontology (e.g. proteins) and the possible relationships between them. The final stage or level is the development of code that can be run by computers (Schuurman, 2006). Ontologies are structured much like a biological taxonomy with general concepts appearing at the top of the tree and becoming more general as one traverses down. The hierarchical schema, however, is only a ‘shell’ that can accommodate the concepts and their relations particular to a domain (Rector, 1999; Schulze-Kremer, 2002). It must be populated by domain knowledge expressed in a formal semantics—a computing syntax such as a markup language—that allows all entities declared into the ontology to be precisely defined and their interrelationships given strict parameters with the goal of enabling realistic biological models.

**Figure 1:**
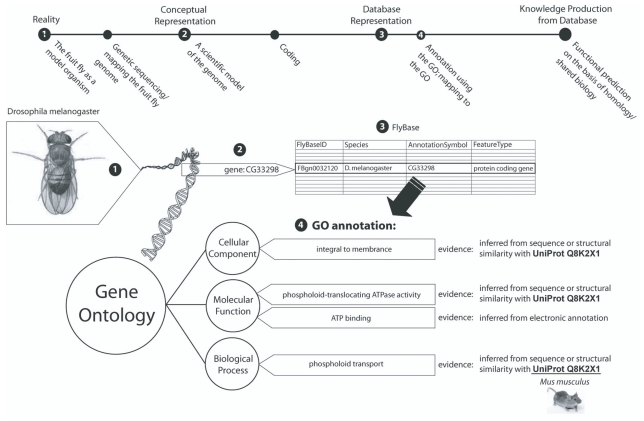
The formalization process. Moving from a concept of a particular gene to its encoded reification and ontological representation. Note how the entity (fruit fly) becomes increasingly represented in digital database format as it is formalized, or abstracted from its real-world form. The entity loses dimensionality, while researchers gain the advantage of computational function. Figure 3 illustrates in more detail the role that entity descriptions—or annotations—play in creating a larger standardized digital knowledge environment for bioinformatics. Note that any gene product many have more than one annotation in the same branch (see Molecular Function this example), and can be annotated in three different branches of GO (Cellular Component, Biological Process, and Molecular Function) (FlyBase 2007, The Gene Ontology Consortium 2077).

Formal semantics permit the distinction of concepts declared into the model (Sowa, 2000). To satisfy the strict criteria of formal ontology building, the formal semantics used to instantiate an ontology should be premised on a formal logics particular to some logical algebra (Smith et al. 2005)—such as description logics (DL)—which contain predetermined rules for “when two concepts are the same, when one is a kind another, or how they differ” (Rector, 1999, p 239–52, p. 10). These rules must furthermore be expressed in some machine-readable syntax—in this case, a knowledge representation language such as the Web Ontology Language (OWL). Such rules govern the expression and processing of *relations* between concepts in the hierarchy. Relational expressions are the implementation basis for all subsequent computing and modeling tasks in a software environment. Figure 1 illustrates the progress from concept to code. Formalization is the basis for the transition from a conceptual entity to a machine-readableform.

The ability to define relationships between concepts distinguishes formal ontologies from earlier integration and interoperability approaches. How they are expressed are detailed in the subsequent sections on the GO and OBO efforts. Relationships are an expression of the *context*—akin to usage in natural language—in which concepts are used or from which they emerge. The utility of capturing relationships between concepts is thus that they convey semantics; content semantics are expressed by identifying how concepts relate to each other in the hierarchical knowledge space. This hierarchical knowledge space is a parent-child structure that conveys the semantic granularity of the relation between any two concepts by rendering entities to be either more specific of more general than each other (Smith et al. 2003). This formal ontological structure implies at least one kind of relation: a hyponymic (*is-a*) relationship is implied by the hierarchical nesting of terms and denoted by their position relative to each other in the family tree on the basis of their subsumption (where a concept is a subclass or member of another) and specialization (where one concept is the superclass of or contains another) (Kohler et al. 2003). Additional relationships can be asserted between concepts as a directional association (i.e. the relationship proceeds from one concept to another). Relationships—referred to as *properties*—are akin to ‘semantic edges’ which depict the meaning of data elements by providing the *context* of their usage (where context is analogous to how concepts participate in class membership).

Formal ontological expressions are stated as propositional triplets consisting of *concepts* (real-world entities that populate the model), their *properties* (or relationships between said entities), and *instances* (particular occurrences of a concept; for example, a particular gene with its own unique identifier in a database) in a hierarchical model (Gardner, 2005; Schulze-Kremer, 2002; Sowa, 2000). A triplet (concept + property + instance) constitutes a proposition, or “[definitive] statement about (part of) the world” (Gardner, 2005; Schulze-Kremer, 2002, p 187). Where an ontology is formal in the sense that it is underwritten on an axiomatic logic such as Desription Logics (DL), the axioms of the logic can be applied to impose *restrictions* that define conditions under which concepts in a domain logically participate in relationships with each other (Gardner, 2005). For example, we can impose a cardinality restriction to specify that, following the series of generic examples provided by Aranguren et al. (2007), a “man” must have at least one testes.

In a strict definition of formal ontology, the axiomatic logics serve to underwrite a formal notation for content specification. For example, DL comprise the logical *semantics* for knowledge representation which constitute the basis of ontological encoding specifically designed for a group of knowledge representation languages which include OWL, the standard language for ontologies over the Web. The eXtensible Markup Language provides the tag-based syntax for OWL, whereas its schema is defined by the Resource Description Framework (RDF), which specifies what the ‘triplet’ structure (*concepts* + *properties* + *instances* described above) of ontological expression. A standard schema ensures that when OWL statements are *parsed* or transformed into the component data structure of the target formal ontology, the parser knows which part of the expression constitutes the concept, which section the relation, and which the instance. It is this structure which makes the grammar of an ontology *meaningful*—in the case of bioinformatics, for example, it anchors annotations to the gene products they characterize (Berman, 2005; Berman and Bhatia, 2005).

This structure moreover makes the ontological model amenable to implementation in a software environment (Smith et al. 2003) in order to allow for the kind of intelligence described using the example of a cardinality restriction in the instance, ‘a man must have at least one tests’. The taxonomic structure of formal ontologies captured using logical notation and expressed in a knowledge representation language allows the semantics of concepts to be computed on the basis of concept inheritance. This is known as *reasoning*, where an application infers non-explicit (not directly stated) relationships between concepts (Rector, 1999). For example, where two proteins identified using different unique identifiers in disparate databases are described as participating in the same biological function, being part of the same sequence, having the same cellular location, etc., they can be recognized as referring to the same concept and can thus be extracted from separate databases on the basis of these functional characteristics rather than nominal IDs. The ability for each term to relate to every other term in the hierarchy is a way of capturing—and expressing—the complexity of biology (Hill et al. 2002). Reasoning can therefore be thought of as supporting both inference and query (Aranguren et al. 2007). Inference consists of computing the hierarchy—for example, it will reveal multiple inheritance amongst classes as mentioned aboved. Query consists of the ability to interrogate the concept hierarchy on the basis of object associations or, conversely, to reveal object associations amongst selected concepts or classes. Imposing the above cardinality restriction therefore has two implications. The first is that any data object labeled or identified “man” in a data repository mapped to an ontology with the above property restriction imposed upon the man-testes relation will be recognized as a (likely person) with at least one testes. Conversely, the execution of reasoning tasks on the ontology or any data structure mapped to it will compute whether all instances of man are consistent with (a person) having at least one testes.

Ontologies—with their hierarchical structures—capture the semantic granularity of biological databases. The property of inheritance allows the computer to process, for example, that the concepts used to annotate two respective sequences are both ‘children’ of the same meta-concepts (i.e. they are a kind or part of a the same overarching concept; alternatively, members of the came class) (Lord et al. 2003). This permits researchers to locate regions of exact correspondence as well as those with a high degree of similarity. Entities may relate but are not synonymous—for example, where ‘protein’ is a subclass of another concept, ‘gene products’ (Ahlqvist, 2004; Ahlqvist, 2005). This does not dictate that proteins and genetic products are one and the same, but rather allows the expression of a membership relation at a much finer semantic resolution such that proteins can be understood as one, but not the sole, kind of gene product (which also includes RNA).

Thus far, we have described the problem of semantic and schematic heterogeneity and introduced ontologies as a means of mitigating the problem. The formal implementation of ontologies—as well as necessary conditions for formality—has also been discussed as well as its advantages for promoting computer reasoning. In the next section, we describe in detail the genesis and development of a bioinformatics portals, GO and its role in biological data interoperability. In addition, we briefly illustrate the implementation of ontologies in two database as well as an ontology-based method for comparing data from different registries or jurisdictions.

## GO: Ontology in Practice

The use of ontologies for bioinformatics is being driven by the proliferation of genome-scale data-sets and the diffusion of the Internet and its protocols for data sharing and exchange (Blake, 2004). Bio-ontologies fulfill two central functions for the biological domain—first, they “clarify scientific discussions” by providing the vocabulary and terms under—and with which—such discussions take place, and second, they enable data discovery across distributed data resources (Blake, 2004, p 773). The pre-eminent bio-ontology is the (GO), a Web-based, open source knowledge resource for bioinformatics and the second-most cited biological data resource after UniProt (Galperin, 2006).

The GO project evolved as a joint endeavor between three model organism databases: FlyBase, Mouse Genome Informatics Database (MGI), and the *Saccharymyces* (yeast) Genome Database in 1999 (Ashburner et al. 2000; Blake, 2004). The formation of the Gene Ontology Consortium (GOC) coincided with the successful completion of the mappings of several eukaryotic genomes. The key to associating these model databases was the genetic structure of organisms (Lewis, 2005). A potential problem lay in that these databases had been designed and populated with competing concepts for gene. Moreover, there was still limited understanding as to how the located genes were controlled and more importantly what functions many of these served (Lewis, 2005). As there is a high degree of functional conservation in homologous organisms, gene function can be reasonably inferred through probable genetic orthologues (Ashburner et al. 2000). In other words, rather than ‘reinventing the wheel’, biologists and bioinformaticians could transfer functional attributes describing the cellular behaviors of gene products between these databases thereby significantly reducing workload.

The chief impediment to this task were not the unique identifiers for the gene products themselves as researchers had been tapping into protein and gene databases such as GenBank and Swiss-Prot, TrEMBL and PIR for decades (the latter three joined to form the Universal Protein or UniProt protein repository in 2002). Because sequences are unique, they could be easily accessed on the basis of sequence characteristics (though there was sequence redundancy between protein repositories). Computationally, because sequences can be quantified, this is a trivial integration task that simply requires the normalization of unique codes (Schuurman et al. 2006). Rather, it was the functional descriptions of gene products that proved challenging. Integration had to proceed within the context of the molecular and biological characteristics of each gene product identified (Lewis, 2005). In an attempt to solve the problem, informatics experts from the three original participating model organism databases devised functional classification systems in the hopes that these precursors to the GO would facilitate interoperability. What soon became apparent, however, was that these functional classifications were not common between organisms (Lewis, 2005).

In other words, the annotation was not consistent from one database to the next. Gene annotation is defined as the “task of adding layers of analysis and interpretation to … raw sequences” (2002, p 755). This includes information about their function, position relative to coding/non-coding boundaries, participating process, etc. (Chicurel, 2002b). Annotations constitute a set of *metadata*, or ‘data about data’ Historically, annotation has been stored as free-text or at best semi-structured descriptions semantically particular to the terminological or classification systems unique to many of the databases (Hill et al. 2002; Lord et al. 2003). There were two challenges. First the use of competing nomenclatures precluded the linear association of database semantics. Second, the expression of these annotations in natural language provided little context for data mining because they were not machine-readable. Returning to the example of functional prediction, protein functions are inherently dependent upon context, particularly cellular context (Carroll and Pavlovic, 2006). This is exacerbated in the case of proteins particularly as many sequences often have multiple functions (Carroll and Pavlovic, 2006).

The GO Consortium formed as a response to the pervasive semantic heterogeneity of biomedical data and its lack of formality. Indeed the GO was designed for making historically free-text based annotations tractable (Lord et al. 2003). The three participating database programs agreed to work in concert to provide the biological community with a consensus-driven framework to guide the annotation of gene products such that their structure (e.g. how molecular function is described and which part of the description occurs in what syntactic order) and semantics (the terms and concepts) are consistent. The result was the GO—a “structured, precisely defined, common, controlled vocabulary for describing the roles of genes and gene products in any organism” (Ashburner et al. 2000, p 26). The GO is not a taxonomy or index of all known proteins and gene products, but rather provides a standardized set of names for genes and proteins and the terms for characterizing—or ‘annotating’—their behaviors (Gene Ontology Consortium 2007).

Gene product semantics are organized into three categories which capture the primary ‘aspects’ of genes: i) biological process, which captures the larger process in which the gene product is active; ii) molecular function, the biochemical function a gene product contributes to that process, and iii) cellular component, the location in the cell where that particular function is fulfilled or expressed (Ashburner et al. 2000; Gene Ontology Consortium 2007; Hill et al. 2002; Lord et al. 2003). Concepts or terms constitute nodes, and vectors referred to as *edges* represent relationships between concepts (Hill et al. 2002; Lord et al. 2003). These three sub-ontologies are maintained independently because the one-to-many relationships between process, function and cellular location would make a singular graph representation intractable (Ashburner et al. 2000). Annotations for the same term in each ‘view’ are cross-referenced on the basis of a unique identifier or serial number assigned to each term in the GO. Increasingly, these identifiers are being used to refer to concepts in other protein and gene-oriented databases and constitute a linear and direct means of mapping databases to the GO (Gene Ontology Consortium 2007). A 2005 figure estimates the GO as consisting of more than 17, 500 terms distributed amongst the three subgraphs (Wolstencroft et al. 2005). All possible annotations for a protein can be represented using these concepts (Carroll and Pavlovic, 2006).

Each of these three separate annotation categories—biological process, molecular function, and cellular component—is represented as its own directed acylic graph, or DAG (Ashburner et al. 2000; Hill et al. 2002; Lord et al. 2003). A DAG is a data structure similar to a tree which represents knowledge hierarchically, mirroring the taxonomic structure of biological knowledge. Any entity can point to any other entity in the mathematical space; this is, however, a direction, and non-recursive, encoding. In other words, concepts can point to other entities in the model, but those entities do not ‘point back’ as in OWL. Indeed the DAG can be considered to be the native knowledge representation (KR) language of the GO (Aranguren et al. 2007). Unlike the KR languages introduced above, however, DAG semantics are not predicated on a formal logic as they are in the case of OWL. Rather, machine readability is instructed by the directional links between pairs of concepts. Semantic ‘edges’ (relationships) in the DAG are simply “ordered pairs of nodes” (Aranguren et al. 2007, p 61). Pointers are like edges in the sense that their semantics are directed, and are labeled with the relationship that associates related classes. These associations are of only two relations: *is-a*, which denotes that concepts are *kinds of* entities, and *part-of*, which can signify the participation or contribution of a concept in a sequence or process (Smith et al. 2003).

The DAG is available in many file formats—XML, OWL—but the most common formal notation in which GO ontologies are rendered is the Open Biomedical Ontology (OBO; described in more detail below) flat file structure which is underwritten by a modified subset of Web Ontology Language (OWL) description logics (DL) concepts for content specification (Gene Ontology Consortium 2007).

Like OWL, OBO is an ontology language, and standard ‘file format’ for GO annotations. It is however less expressive than OWL. These relations are unidirectional and linear as per the DAG data model and do not require the recursive relational declarations (where the reciprocal or inverse of a relationship is also encoded) characteristic of OWL statements. Thus a flat file structure that only supports sequential reading is appropriate for the GO because relations are read from broader or general to more specific or precise concepts.

At the level of the database, the GO is represented as a structured vocabulary; more specifically, as gene product annotations expressed using concepts and their tripartite (biological, molecular, and cellular) structure defined in the GO (Hill et al. 2002). The GO is not considered an informatics ontology in the full sense of the term because it has not been designed to be deployed within software environments which execute semantic inference on the basis of logical semantics (Smith et al. 2003). Moreover it does not fulfill the conditions of formality identified by Smith et al. (2005). Rather it is considered and referred to by its engineers as a “controlled vocabulary” (Ashburner et al. 2000, p 26). The nevertheless has many of the characteristics of a formal ontology: machine-readability, formal notation, a hierarchical knowledge structure, and relational associations between concepts. In other words, the GO may be considered a partial implementation that uses many concepts of formal ontology. Part of the reason, however, that the GO is only a partial implementation is that it was designed to be operational within existing infrastructures, requiring no changes to existing architectures.

Notwithstanding, the GO provides the standard vocabulary for semantic integration and automated tasks for bioinformatics. As such it is more than merely a sophisticated data dictionary. Whereas controlled vocabularies or data dictionaries provide a definition of the terms used by a community of practice and these may indeed be machine-readable and thereby formal, a nomenclature does not capture the hierarchical representation of knowledge nor the corresponding relations between all concepts in the data space, and thereby does not support computational reasoning (Schulze-Kremer, 2002). Several terminological systems such as SNOMED (Systematized Nomenclature for Medicine) and MeSH (Medical Subject Headings) have, however, been mapped to the GO (Searls, 2005).

**Figure 2:**
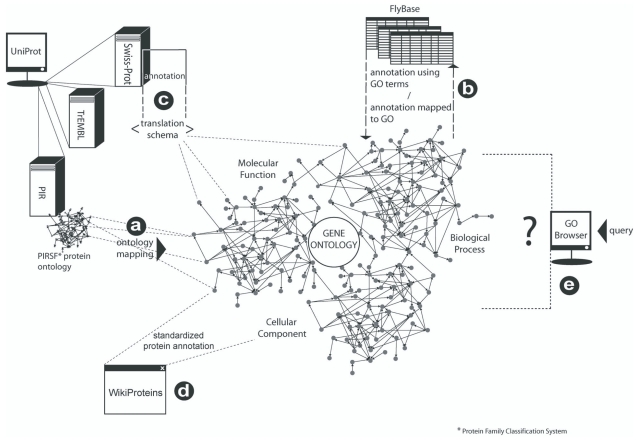
The Gene Ontology as a global ontology for bioinformatics. Smaller scale bioinformatics ontologies almost invariably map to the GO (**a**). Several large databases, such as FlyBase (**b**), contribute annotation to the GO using its semantics such that there is a direct mapping between genes/gene products at the database level and their participation in the ontology. (FlyBase annotation is explained in greater detail in Fig. 1). Where annotation is unique to the database, a translation program can transform annotation into a tractable GO representation (**c**) (Camon et al. 2004). The GO provides a standardized vocabulary for the description of genes and gene product across not only databases but also in emerging bioinformatics infrastructures, such as WikiProteins (**d**). The consistency of semantics reduces ambiguity in the query of bioinformatics resources, and allows genes and gene products to be retrieved on the basis of common biology rather than lexical coincidence (**e**).

The GO is a *global ontology* (Lord et al. 2003). In other words, it is a central knowledge proxy to which other ontologies or knowledge representations may be aligned. Ontology mapping is the process of defining associations between ontologies. This involves the formal declaration of relational links between entities, much like that involved in relating concepts in a hierarchical ontological structure. Ontologies can either be *aligned* whereby the formalisms remain separate entities but are related, or *merged* wherein a singular ontology is generated from the crossproducts of two input ontologies (Choi et al. 2006). ‘Mapping’ is thus unidirectional and always *from* the constituent database *to* the GO. Figure 2 illustrates the role that GO plays in development of global biological ontology and the mechanics involved.

A global ontology paradigm is appropriate for the domain because there is a finite, though as yet not fully discovered or known, body of genetic information shared between all life on Earth (Ashburner et al. 2000). Accordingly there is no need to build *local* ontologies each capturing a competing account or version of a biological universe. Such a scenario would be much more intensive, requiring the definition of linkages between each participating ontology. A global ontology serves as a *proxy context* which interfaces all participating knowledge formalisms are translated to the unique semantic points of the proxy and then compared on the basis of this translation (Ahlqvist, 2005).

The alignment of currently non-compatible ontologies to the GO is one avenue for its *curation* or the process of developing and contributing content or adding value to digital knowledge representation systems such as databases or ontologies. For the GO to serve as a comprehensive knowledge resource for the biological community, it must reflect the continuously increasing body of biological, specifically genetic-level, knowledge. In other words, it must expand to keep pace with the identification of new genes, sequences, functional determinations, etc. Rather than being the responsibility of the Consortium, GO curation has been user driven from inception (Hill et al. 2002). GO expansion efforts are supported by the scientific publication process, with several leading periodicals and sequencing initiatives mandating that newly identified sequences be deposited into GO-compliant databases and any new annotations be added to the GO (Chicurel, 2002b; Peters and Sette, 2007). Early curation was characteristically on a need-be basis with concepts added to the GO when authors were annotating genes, etc. (Hill et al. 2002). Such a*d hoc* practices, however, resulted in logical problems in the DAG and indeed soon became inefficient as the scope and scale of the GO has steadily grown (Hill et al. 2002). Increasingly, methods for contributing annotations to the GO are based on the automatic generation of annotation concept definitions on the basis of cross-products between databases (as local ontologies) and the GO itself (Hill et al. 2002).

The GO was designed specifically to account for molecular function, biological process, and cellular components of gene products. It lacks the semantics to describe the physical attributes of genes, to describe a protein family, or to account for experimental processes and diagnostic procedures (Wolstencroft et al. 2005). There are both proprietary and open ontologies with richer semantics for more specific description tasks for biology either being developed or presently available (for examples, see Chicurel, 2002; Peters and Sette, 2007; Schulze-Kremer, 2002). The majority, however, are designed with mapping to the GO in mind (Buckingham, 2004b; Chicurel, 2002b).

## Open Biomedical Ontologies

GO is thus not the sole ontology for biology. Indeed there is a need for ontologies to parallel the GO programme. The GO is only one—but certainly the most prominent—ontology effort which contributes to the Open Biomedical Ontologies (OBO) initiative (Gene Ontology Consortium 2007). The OBO Foundry is an umbrella for over 60 bio-ontologies (Smith et al. 2007, The Open Biomedical Ontologies 2007). It provides guidelines for ontology development, and indeed ontologies such as GO have been restructured in line with OBO specifications. As indicated above in the detailed discussion of GO, OBO is also its own ontology format (although OBO does provide an extensive suite of translation schema for mapping OBO representations to, for example, OWL) (The Open Biomedical Ontologies 2007). The benefit of this is that, given domain consensus, it provides for uniform representation and thereby increased interoperability. For example, disparate cell-type ontologies including the GO are now integrated into a single ontology that is itself being aligned to a singular implementation. OBO participates in the National Center for Biomedical Ontology and is slated to become a centralized resource of its emergent BioPortal in support of bioinformatics knowledge discovery and sharing.

## Ontologies in Support of Bioinformatics

The largest public contributor of annotations to the GO project is the Gene Ontology Annotation Database (GOA) (Camon et al. 2004). While annotation is the central organizing principle and *raison d’etre* of the GO, the potential of their ontological encoding is not to have a hierarchically structured record of concepts used to annotate the data of biology, but rather to exploit the ontology for a series of bioinformatics services which remove the burden of data-intensive tasks from molecular biologists and moreover *produce* knowledge over and above facilitating its reuse.

One of the primary objectives for bioinformatics to realize is the automation of annotating cross-matches between databases (Ashburner et al. 2000). The electronic generation of annotations based on homology is particularly desirable as the manual curation of gene-oriented databases is time consuming and non-trivial for humans (Chicurel, 2002b). The GO facilitates the automatic annotation of gene products at the database level. GOA for instance uses GO terms to generate annotations for the UniProt Knowledgebase (The consortium of SwissProt, TrEMBL, and PIR-PSD protein databases) (Camon et al. 2004). Existing data held in UniProt are electronically associated with or translated into GO terms on the basis of a defined mapping file used to facilitate the conversion of keywords in the constituent databases to tractable GO representations (Camon et al. 2004). Once the semantics are consistent between data sources, biologists who have identified a new sequence, for example, can navigate the GO via an interface known as an *ontology browser* on the basis of these common data elements and indeed use the existing GO annotations to not only discover sequence similarity but to also automatically populate or their own database using the existing annotations for homologues from other curated data sources. Thus the ontology functions as a ‘translation schema’ (Buetow, 2005). This is possible because GO is underwritten by a structured grammatical framework (e.g. RDF) that predetermines the occurrence or sequence of description types in a proposition, allows the expression to be parsed and correctly broken-down such that it can be stored according to the structure of the target database.

The GO can be used to automate the following services: database annotation, GO extension (automating the transfer of new annotation concepts *to* the GO), prediction services, and database population. Prediction services supported by ontologies yield new biological knowledge. Gene location using current generation algorithms uses data from a pair of genomes to locate areas of genetic affinity; these areas of ‘overlap’ are often the sites of new genes (Chicurel, 2002b). The success of this is based on the semantic consistency of annotations for the input genomes. In addition, the GO supports nuanced data exploration and query (Blake, 2004). The hierarchical structure of knowledge afforded by ontology allows the isolation of the appropriate concept for query on the basis of its context or position relative to other entities in the data space (Buckingham, 2004b). This allows users to formulate searches using conventional keywords, but resolves the meanings of those keywords.

Once the protein or gene of interest is isolated, its location confers more information than a binary indication of its absence or presence in a database. Not only do we know about the occurrence of a protein, for example, but we are told something *about* it. The proprietary EnsemblGO Browser is an interface which compiles annotations to generate reports or summaries centered on the biological entities isolated in the GO such that, for instance, “the previously unconnected classes Antigen, Immunogen, and Adjuvant are now recognized as being objects (for example, Proteins), which participate in a certain role (as Immunogens) in a specific process (such as Immunization)” (Buckingham, 2004b; Peters and Sette, 2007, p 489).

Ontologies can further be used as a basis for exploring datasets. We have devised a methodology called *ontology-based metadata* which uses ontologies as a component in a metadata-based framework for the comparison of a series of eight ‘near’ but non-equivalent terms that have been identified as an obstacle to integrating perinatal (pregnancy and antepartum) health data registries across Canada. Our objective is to provide health researchers and data stewards with a basis for drawing meaningful parallels between data elements to enable the legitimate integration of peri-natal data registries. Ontology-based metadata for each term is first collected via a series of electronic forms which standardize the description of each concept. Each constituent database is responsible for detailing how these terms are *used* in their particular jurisdiction—or context. This includes a specification of the classification standard used (e.g. ICD-10), the identification of thresholds for measurement specifications, and space for free-text descriptions of any policy constraints which may influence how the term is used in a given jurisdiction. In addition to these ‘annotations’, we encode each perinatal database as a formal ontology in OWL. These ontologies capture the semantic structure of database terms. These are then merged into a single ontology, with the relationships between each and every concept defined in the product tree. Both the ontology-based metadata and the ontologies are inputs to a semantic data discovery portal where researchers specify which terms in two respective databases are to be compared via a graphical user interface (GUI). The application returns to the user both the encoded relationship between the concepts extracted from the OWL code—for example, where *pregnancy-induced hypertension* is a *KIND-OF hypertension complicating pregnancy*—and the ontology-based metadata for each term in the selected databases. Thus the researcher is provided with both a marker for the granularity of the semantic relationship between two concepts, as well as valuable metadata which are used to inform perinatal database decisions.

**Figure 3:**
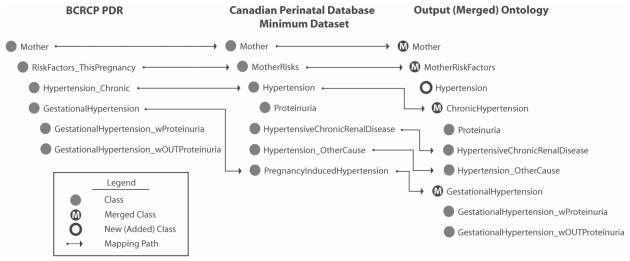
Ontology mapping. An ontology for hypertension resulting from the merging of hypertension concepts in the British Columbia Reproductive Care Program Perinatal Database Registry (BCRCP PRD) and the Canadian Perinatal Database Minimum Dataset. The resulting output ontology shows the hirerarchical nesting of hypertension semantics originating in respective databases in relation to each other.

The gestational hypertension/hypertension example above would indicate that hypertension experienced during pregnancy is a more general concept which includes gestational hypertension but also encompass pre-existing hypertension. In some databases, hypertension and pregnancy-induced or gestational hypertension are not differentiated from chronic or pre-existing incidences of disease. Alternatively, in other databases, these concepts are distinguished from each other on the basis of the periodicity of disease onset such that chronic hypertension and pregnancy-induced or gestational hypertension are disjoint (database *A*). In yet other registries, any form of hypertension presenting during pregnancy is considered gestational such that a pre-existing condition which first manifests itself during pregnancy is still encoded as pregnancy-related (database *B*). There is thus a semantic incommensurability between what ‘gestational hypertension’ represents in databases *A* and *B*, precluding a direct mapping between these concepts indicating semantic equivalence. Rather, ‘gestational hypertension’ in database *A* would be a *kind of* gestational hypertension as the concept is reified in database *B*. If a researcher were to query ‘gestational hypertension’ across both databases, she would logically accept them as referring to the same concept on the basis of lexical coincidence. However, the lack of an encoded equivalence between these two concepts would preclude their conflation. Thus our this approach not only provides information regarding how concepts should be associated, but also uses formal ontologies to restrict *which* concepts may be legitimately compared. This nesting of relationships between semantic terms is described in Figure 3.

Another instantiation of the ontology-based metadata concept similar to our implementation is WikiProteins, a structured semantic space for capturing the context—biological, physiological, chemical, etc.—of proteins and then sharing that collaborative knowledge with other biologists in real-time (Giles, 2007; Wiki For Professionals 2007). Historically, the problem with metadata has been that it is so labor intensive and never updated (Schuurman and Leszczynski, 2006). WikiProteins provides a mechanism for sharing the labor and ongoing maintenance by participants. This collaborative Web-based workspace facilitates the open curation of protein-specific information by providing biologists and bioinformaticians with a means of contributing to the cumulative body of biological knowledge. At the moment, it serves UniProt and GO descriptions for the annotation of proteins via a series of standardized forms or ‘slots’ for their description. This consists of definitions, attribute-value relations (e.g. a protein can be given the attribute “tissue” with the value “[e]xpressed in muscle fibers”), and provisions for disambiguating sequences or instances of proteins by identifying synonyms, disjoint concepts, alternate spellings, etc. (Wiki For Professionals 2007). Curators can link their descriptions or proteins to other citations, references, and publications indexed in PubMed. Moreover, the wiki concept ensures that these annotations are self-validating. Other users can go in and add or revise the annotations. For example, using the “tissue” example above, a subsequent curator can reify this protein as “[e]xpressed in muscle fibers and *the brain*” (Wiki For Professionals 2007). Similar to our ontology-based metadata approach, it combines both free-text fields for open description and more restrictive means of disambiguating proteins and protein concepts. For instance, it extends the ability to identify whether these synonyms are instances of equivalent meaning, or if they are different. If the latter is the case, curators can further annotate—or describe—specifically where these differences lie. WikiProteins is but one example of where the GO is being deployed to provide a standardized vocabulary for annotation across distributed data resources.

As our non-automated method for data discovery and WikiProteins for protein knowledge exchange illustrate, ontologies are not standalone solutions for interoperability but rather comprise a component of or input to large-scale interoperability infrastructures. Indeed ontologies are knowledge representations and *not* software applications, having no innate functionality. As such they must be deployed within digital architectures where constituent programs can exploit the hierarchical structure of formal ontologies to facilitate data sharing at the level of semantics. Many such cyber-infrastructures exist for biology and biomedicine. A notable example is the Cancer Biomedical Informatics Grid (caBIG), a Web-based National Institutes of Health (NIH) data consortium for cancer research (National Institutes of Health 2007). caBIG is built on an open grid architecture similar to a federated database environment where users are presented with a central interface which seamlessly integrates participating databases, but with the addition of Web services that provide tools and applications. The emphasis of caBIG is on the provision of services—such as data analysis tools, applications, scripts, algorithms, etc.—relevant to cancer research. The grid is organized into a series of “workspaces” or virtual communities where participants can both access, revise, and upload new technologies to that specific sub-domain of application or interest. The emphasis of caBIG is on services, with participants notifying each other of the constituent services they make available by means of UML (Unified Modeling Language) metadata wherein the services are described using standardized DL-annotated concepts from a vocabulary service which defines terms and concepts in biomedical vocabularies. Here, ontologies are utilized as a standardized set of concepts and terms across applications and services for their uniform description such that researchers can locate and access the appropriate technologies on a need-be basis. This provides interoperability across distributed cancer research centers at the level of services.

## Conclusion

We have described the challenges inherent in semantic integration of biological databases. Many of these are common to all semantic integration realms and are based on the problem of language not being transparent across institutional and user environments. In biology and other disciplines, ontologies or strict paradigmatic taxonomies have been used to mitigate the problems associated with semantic integration. Ontologies are a means of conveying context associated with semantic terms so that their meaning is transparent between multiple data users. This paper has described in depth the use of ontologies for data integration in biology. Biology has led the scientific world in developing a number of unique approaches to semantic integration. These unique ontology-based integration efforts include ontologies such as the Gene Ontology (GO), and frameworks that exploit their machine-readable semantics to support bioinformatics tasks, such as the cancer Biomedical Informatics Grid (caBIG) and the incipient WikiP-roteins knowledge community. In addition, we introduced the concept and early implementation of ontology-based metadata in order to demonstrate the role that context plays in clarifying hierarchical and schematic relationships between like but non-equivalent semantic terms in different databases. Each of these is a unique approach to surpassing the problems associated with a lack of congruity in language and meaning across scientific databases.

## Brief Glossary

**Ontology** has a different meaning in philosophy than computing. In the former, it means the essence of being. In computing and information sciences, an ontology is a formal universe in which each entity is precisely defined and its relationship with every other entity in the specific categorical or computing realm is precisely determined. Ontologies in this context are the range of what is possible—in a computing context. They can be thought of as simply a classification system, a map legend, or a data dictionary.

**Epistemology** is the study of “how we know what we know.” In other words, epistemology is the lense through which we view reality. Epistemology refers, in the broadest sense, to the methods that we use to study the world and the perspective that a researcher uses to interpret entities and phenomena.

**Semantics** refers to the ways in which language is interpreted differently in different context, environments or in different institutional cultures.

**Gene prediction**—also referred to as gene finding—uses algorithms to identify biologically functional regions—or exons—of sequences which explicitly code for proteins. These are referred to as coding regions.

**Gene mapping** is the creation of a genetic map in which DNA fragment are linked to chromosomes.

## References

[b1-bbi-2008-187] Agarwal P 2005 Ontological considerations in GIScience International Journal of Geographical Information Science 19 501 36

[b2-bbi-2008-187] Ahlqvist O 2004 A parametrized representation of uncertain conceptual spaces Transactions in GIS 8 493 514

[b3-bbi-2008-187] Ahlqvist O 2005 Using uncertain conceptual spaces to translate between land cover categories International Journal of Geographical Information Science 19 831 57

[b4-bbi-2008-187] Aranguren ME Bechhofer S Lord P Sattler U Stevens R 2007 Understanding and using the meaning of statements in a bio-ontology: recasting the Gene Ontology in OWL BMC Bioinformatics 8 57 69 17311682 10.1186/1471-2105-8-57 PMC1819394

[b5-bbi-2008-187] Ashburner M Ball CA Blake JA 2000 Gene Ontology: Tool for the unification of biology Nature Genetics 25 25 9 10802651 10.1038/75556 PMC3037419

[b6-bbi-2008-187] Baader F Horrocks I Sattler U Staab S Studer R 2004 Description logics Handbook on ontologies Berlin Springer-Verlag 3 28

[b7-bbi-2008-187] Berman JJ 2005 Pathology data integration with extensible markup language Human Pathology 36 139 45 15754290 10.1016/j.humpath.2004.10.013

[b8-bbi-2008-187] Berman JJ Bhatia K 2005 Biomedical data integration: Using xml to link clinical and research data sets Expert Review of Molecular Diagnostics 329 36 15934811 10.1586/14737159.5.3.329

[b9-bbi-2008-187] Bisby FA 2000 The quiet revolution: Biodiversity informatics and the internet Science 289 2309 11 11009408 10.1126/science.289.5488.2309

[b10-bbi-2008-187] Blackmore S 2002 Biodiversity update—progress in taxonomy Science 298 365 12376687 10.1126/science.1075026

[b11-bbi-2008-187] Blake J 2004 Bio-ontologies—fast and furious Nature Biotechnology 22 773 4 10.1038/nbt0604-773 15175701

[b12-bbi-2008-187] Blake JA Bult CJ 2006 Beyond the data deluge: Data integration and bio-ontologies Journal of Biomedical Informatics 39 314 20 16564748 10.1016/j.jbi.2006.01.003

[b13-bbi-2008-187] Boguski MS McIntosh MW 2003 Biomedical informatics for proteomics Nature 422 233 7 12634797 10.1038/nature01515

[b14-bbi-2008-187] Bowker GC 2000 Mapping biodiversity International Journal of Geographical Information Science 14 739 54

[b15-bbi-2008-187] Buckingham S 2004a Data’s future shock Nature 428 774 7 10.1038/428774a 15085138

[b16-bbi-2008-187] Buckingham S 2004b Getting the meaning Nature 428 776 15085141 10.1038/428776a

[b17-bbi-2008-187] Buckingham S 2007 To build a better model Nature Methods 4 367 74

[b18-bbi-2008-187] Buetow KH 2005 Cyberinfrastructure: empowering a “third way” in biomedical research Science 308 821 4 15879210 10.1126/science.1112120

[b19-bbi-2008-187] Camon E Magrane M Barrell D 2004 The gene ontology annotation (GOA) database: Sharing knowledge in uniprot with gene ontology Nucleic Acids Research 32 D262 D6 14681408 10.1093/nar/gkh021 PMC308756

[b20-bbi-2008-187] Carroll S Pavlovic V 2006 Protein classification using probabilistic chain graphs and the gene ontology structure Bioinformatics 15 1871 8 16705013 10.1093/bioinformatics/btl187

[b21-bbi-2008-187] Castro AG Rocca-Serra P Stevens R 2006 The use of concept maps during knowledge elicitation in ontology development processes—the nutrigenomics use case BMC Bioinformatics 267 80 16725019 10.1186/1471-2105-7-267 PMC1524992

[b22-bbi-2008-187] Chicurel M 2002a Bioinformatics: Bringing it all together Nature 419 751 7 10.1038/419751a 12384707

[b23-bbi-2008-187] Chicurel M 2002b Putting a name on it Nature 419 755 7 12384708 10.1038/419755a

[b24-bbi-2008-187] Choi N Song IY Hyoil H 2006 A survey on ontology mapping SIGMOD Record 35 34 41

[b25-bbi-2008-187] FlyBase 2007 Flybase: A database of *drosophila* genes and genomes, version fb2007_01 [online] Accessed 04 September 2007 http//flybase.bio.indiana.edu/: http://flybase.bio.indiana.edu/

[b26-bbi-2008-187] Galperin MY 2006 The molecular biology database collection: 2006 update Nucleic Acids Research 34 D3 D5 16381871 10.1093/nar/gkj162 PMC1347524

[b27-bbi-2008-187] Gardner SP 2005 Ontologies and semantic data integration Drug Discovery Today 10 1001 7 16023059 10.1016/S1359-6446(05)03504-X

[b28-bbi-2008-187] Gene Ontology Consortium 2007 The gene ontology [online] Accessed 15 July 2007 http://www.geneontology.org: http://www.geneontology.org

[b29-bbi-2008-187] Giles J 2007 Key biology databases go wiki Nature 445 691 17301755 10.1038/445691a

[b30-bbi-2008-187] Hill DP Blake JA Richardson JE 2002 Extension and integration of the gene ontology (go): Combining go vocabularies with external vocabularies Genome Research 12 1982 91 12466303 10.1101/gr.580102 PMC187579

[b31-bbi-2008-187] Kohler J Philippi S Lange M 2003 SEMEDA: Ontology based semantic integration of biological databases Bioinformatics 18 2429 7 10.1093/bioinformatics/btg340 14668226

[b32-bbi-2008-187] Kohler J Schulze-Kremer S 2002 The semantic metadatabase (SEM-EDA): Ontology based integration of federated molecular biological data sources In Silico Biology 2 219 31 12542408

[b33-bbi-2008-187] Lewis SE 2005 Gene ontology: Looking backwards and forwards Genome Biology 6 103.1.4 15642104 10.1186/gb-2004-6-1-103 PMC549054

[b34-bbi-2008-187] Lord PW Stevens RD Brass A 2003 Semantic similarity measures as tools for exploring the gene ontology Pacific Symposium Biocomputing Lihue, Hawaii 10.1142/9789812776303_0056 12603061

[b35-bbi-2008-187] National Institutes of Health 2007 Cancer biomedical informatics grid [online] Accessed 10 August 2007 https://cabig.nci.nih.gov/

[b36-bbi-2008-187] The Open Biomedical Ontologies 2007 The Open Biomedical Ontologies [online] Accessed 24 December 2007 http://obofoundry.org/about.shtml

[b37-bbi-2008-187] Pennisi E 2000 Taxonomic revival Science 289 2306 8 11041799 10.1126/science.289.5488.2306

[b38-bbi-2008-187] Peters B Sette A 2007 Integrating epitope data into the emerging web of biomedical knowledge resources Nature Reviews Immunology 7 485 90 10.1038/nri2092 PMC7097317 17479127

[b39-bbi-2008-187] Rector AL 1999 Clinical terminology: Why is it so hard? Methods of Information Medicine 38 239 52 10805008

[b40-bbi-2008-187] Saeys Y Rouze P Van de Peer Y 2007 In search of the small ones: Improved prediction of short exons in vertebrates, plants, fungi and protists Bioinformatics 23 414 20 17204465 10.1093/bioinformatics/btl639

[b41-bbi-2008-187] Sauer U Heinemann M Zamboni N 2007 Genetics: Getting closer to the whole picture Science 316 550 1 17463274 10.1126/science.1142502

[b42-bbi-2008-187] Schulze-Kremer S 2002 Ontologies for molecular biology and bioinformatics In Silico Biology 2 179 93 12542404

[b43-bbi-2008-187] Schuurman N 2006 Why formalization matters: Critical GIS and ontology research Annals of the Association of American Geographers 96 726 39

[b44-bbi-2008-187] Schuurman N Leszczynski A 2006 Ontology-based metadata Transactions in GIS 10 709 26

[b45-bbi-2008-187] Schuurman N Leszczynski A Fiedler R Riedl A Kainz W Elmes G 2006 Building an integrated cadastral fabric for higher resolution socioeconomic spatial data analysis Process in spatial data handling: 12th international symposium on spatial data handling Berlin Heidelberg New York Springer 897 920

[b46-bbi-2008-187] Searls DB 2005 Data integration: Challenges for drug discovery Nature Reviews Drug Discovery 4 45 58 10.1038/nrd1608 15688072

[b47-bbi-2008-187] Smith B Ashburner M Rosse C Bard J Bug W Seusters W Goldberg LJ Eilbeck K Ireland A Mungall CJ Leontis N Rocca-Serra P Ruttenber A Sansone S-A Sheuermann RH Shah N Whetzel PL Lewis S the OBO Consortium 2007 The OBO Foundry: coordinated evolucation of ontologies to support biomedical integration Nature Biotechnology 25 1251 5 10.1038/nbt1346 PMC2814061 17989687

[b48-bbi-2008-187] Smith B Ceusters W Klagges B Kohler J Kumar A Lomax J Mungall C Neuhaus F Rector AL Rosse C 2005 Relations in biomedical ontologies Genome Biology 6 R.46 10.1186/gb-2005-6-5-r46 PMC1175958 15892874

[b49-bbi-2008-187] Smith B Williams J Schulze-Kremer S Musen M 2003 The ontology of the gene ontology AMIA 2003 Annual Symposium Proceedings Washington, DC PMC1480173 14728245

[b50-bbi-2008-187] Sowa JF 2000 Knowledge representation: Logical, philosophical, and computational foundations Pacific Grove, CA Brooks/Cole

[b51-bbi-2008-187] Stanford Medical Informatics 2005 The protege ontology editor and knowledge acquisition system

[b52-bbi-2008-187] Sugden A Pennisi E 2000 Diversity digitized Science 29 2305 11041798

[b53-bbi-2008-187] Thomas CE Ganji G 2006 Integration of genomic and metabonomic data in systems biology—are we ‘there’ yet? Current Opinion in Drug Discovery and Development 9 92 1000 16445121

[b54-bbi-2008-187] Wang K Tarczy-Hornoch P Shaker R 2005 Biomediator data integration: Beyond genomics to neuroscience data AMIA Annual Symposium AMIA PMC1560529 16779146

[b55-bbi-2008-187] Wiki For Professionals 2007 Wikiproteins [online] Accessed 24 July 2007 http://wikiprofessional.info: http://wikiprofessional.info

[b56-bbi-2008-187] Wolstencroft K McEntire R Stevens R 2005 Constructing ontology-driven protein family databases Bioinformatics 25 1685 92 15564301 10.1093/bioinformatics/bti158

